# Change in Hemoglobin Was Not a Reliable Diagnostic Screening Test for Postpartum Hemorrhage: A French Prospective Multicenter Cohort Study (HERA Study)

**DOI:** 10.3390/healthcare11081111

**Published:** 2023-04-13

**Authors:** Chloé Barasinski, Marine Pranal, Stéphanie Léger, Anne Debost-Legrand, Françoise Vendittelli

**Affiliations:** 1 Université Clermont Auvergne, CHU, CNRS, SIGMA Clermont, Institut Pascal, F-63000 Clermont-Ferrand, France; 2 Laboratoire de Mathématiques UMR CNRS 6620, Université Blaise Pascal, F-64170 Aubière, France; 3 Réseau de Santé en Périnatalité d’Auvergne, Pôle Femme et Enfant, Centre Hospitalier Universitaire, Site Estaing, 1 Place Lucie et Raymond Aubrac, F-63003 Clermont-Ferrand CEDEX 1, France; 4 AUDIPOG (Association des Utilisateurs de Dossiers Informatisés en Pédiatrie, Obstétrique et Gynécologie), RTH Laennec Medical University, 7 Rue Guillaume Paradin, F-69372 Lyon CEDEX 08, France

**Keywords:** blood loss estimation, hemoglobin change, postpartum hemorrhage, screening test, vaginal delivery

## Abstract

Changes between pre- and postpartum hemoglobin might be useful for optimizing the postpartum diagnosis of postpartum hemorrhage (PPH), defined as a blood loss exceeding 500 mL. This study’s principal objective was to estimate the mean change in hemoglobin (between pre/post-delivery hemoglobin) among women with vaginal deliveries and PPH. The secondary objectives were to analyze: hemoglobin changes according to blood volume loss, the appropriateness of standard thresholds for assessing hemoglobin loss, and the intrinsic and extrinsic performances of these threshold values for identifying PPH. French maternity units (*n* = 182) participated in the prospective HERA cohort study. Women with a vaginal delivery at or after a gestation of 22 weeks with a PPH (*n* = 2964) were eligible. The principal outcome was hemoglobin loss in g/L. The mean hemoglobin change was 30 ± 14 g/L among women with a PPH. Overall, hemoglobin decreased by at least 10% in 90.4% of women with PPH. Decreases ≥ 20 g/L and ≥40 g/L were found, respectively, in 73.9% and 23.7% of cases. Sensitivity and specificity values for identifying PPH were always <65%, the positive predictive values were between 35% and 94%, and the negative predictive values were between 14% and 84%. Hemoglobin decrease from before to after delivery should not be used as a PPH diagnostic screening test for PPH diagnosis for all vaginal deliveries.

## 1. Introduction

Postpartum hemorrhage (PPH) remains a major cause of maternal deaths worldwide [1,2]. In France, the maternal mortality ratio from 2013 through 2015 was 10.8 deaths per 100,000 live births (95% confidence interval (CI), 9.5 to 12.1) [3]. Obstetric hemorrhage was responsible for 8.4% of French maternal deaths in that period [3]. Hemorrhage is among the most preventable causes of maternal death. Expert assessment has estimated that 57.8% of French maternal deaths were considered preventable or possibly preventable, and in 66% of cases the care provided was not optimal [3]. Obstetric hemorrhage events are also the main cause of immediate or long-term maternal morbidity: respiratory distress syndrome, renal failure, coagulopathy, shock, myocardial ischemia, hysterectomy and other surgical procedures, uterine necrobiosis after radiologic artery embolization, sterility, etc. [4,5,6,7]. Anemia in the postpartum period can cause fatigue, infections, breathlessness, cardiovascular disease, hypopituitarism, maternal stress, anxiety, and depression—all compromising the mother–child bond [7,8,9,10,11].

The prevalence of PPH varies widely throughout the world [12], linked in part to different definitions of PPH and different methods of measuring blood loss (visual estimation, direct measurement, gravimetry, photometry, and miscellaneous methods) [13,14]. The prevalence levels of PPH (defined as blood loss > 500 mL) and severe PPH (defined as blood loss > 1000 mL) are reported to be around 6.0% and 1.9%, respectively, of all deliveries [12]. In a French prospective population-based study, PPH incidence after vaginal delivery was 3.36% (95%CI, 3.25% to 3.47%) and after cesareans (>1000 mL) 2.83% (95%CI, 2.63% to 3.04%). The incidence of severe PPH after vaginal delivery (>1000 mL) was 1.11% (95%CI, 1.05% to 1.18%) and after cesareans (>1500 mL) 1.00% (95%CI, 0.88% to 1.13% [6].

Studies have shown that the visual estimation of blood loss is imprecise and subjective, and tends to overestimate blood loss for small volumes and underestimate it for large volumes [15]. However, visual estimates are widely used throughout the world as the first-line method for diagnosing PPH. The scientific evidence is insufficient to support the use of one method over another for blood loss estimation after vaginal birth [16]. Even though poor maternal outcomes with PPH are generally attributed to delays in the diagnosis and management of PPH, there is little scientific evidence that maternal outcomes can be improved by more accurate measurements of the blood volume loss [17].

It has also been suggested that, because some women giving birth have undiagnosed immediate PPH, changes between pre- and postpartum blood counts might be useful for optimizing PPH diagnosis in the postpartum period [18,19,20,21,22]. Among these studies, one noted that hemoglobin measurement after elective cesarean delivery in asymptomatic women at low risk is unnecessary (*n* = 383; mean loss 13.7 ± 8.7 g/L of hemoglobin) [21]. Two found a weak association between intrapartum blood loss and B-hemoglobin determined at 3 days and 10 weeks after delivery (*n* = 634) [20], or between 12 h and 3–5 days after delivery in a secondary analysis of three randomized trials [22], while others have reported that the peripartum change in hemoglobin level was useful for screening women with undiagnosed PPH after giving birth [18,19]. Among these studies, a few were prospective, and most were limited by the small sample size for the PPH group [18,19,20]. A few studies included cesarean deliveries [21]. The studies included in the publication by Anger et al. were heterogeneous regarding the methods of objective blood loss measurement and the timing of pre- and post-delivery hemoglobin evaluation [22].

The hypothesis of our study was that if a hemoglobin change value makes it possible to identify an undiagnosed PPH, this difference should be clearly visible among women with a diagnosed immediate PPH.

The principal objective of this study was therefore to estimate the mean change in hemoglobin value (variation between pre- and post-delivery hemoglobin levels) among women who had a PPH after vaginal delivery (PPH > 500 mL). The secondary objectives were to: analyze hemoglobin change as a function of the blood volume loss (mild PPH >500 mL and <1000 mL; severe PPH ≥ 1000 mL), analyze the appropriateness of standard thresholds for assessing hemoglobin losses (≥20 g/L, ≥40 g/L, and >10%), and study the performance—both intrinsic (sensitivity and specificity) and extrinsic (positive and negative predictive values)—of the hemoglobin change thresholds selected to identify PPH.

## 2. Materials and Methods

### 2.1. Study Design and Setting

The HERA project was a multifaceted French study including a prospective multicenter cohort study to assess the incidence of PPH and 3 cross-sectional studies. The design and results have been published elsewhere [6,23,24,25,26]. This prospective cohort study is a secondary analysis of the main HERA cohort study, which included all women with an immediate PPH after giving birth in a large number of French maternity wards [6]. A French institutional review board (Comité d’Ethique des Centres d’Investigation Clinique de l’Inter-Région Rhônes-Alpes-Auvergne, Grenoble: CECIC: IRB 0917) approved the HERA study on 9 November 2009. The database was reported to the French Data Protection Authority (CNIL: Commission Nationale de l’Informatique et des Libertés) with report number 1463802 on 7 December 2010.

### 2.2. Selection of Participants

From 11 February through 31 July 2011, 182 French maternity units participated in the prospective HERA study. During this period there were 129,110 deliveries in the 182 participating maternity units (103,733 vaginal and 25,377 cesarean) with 4207 PPH reported, including 3488 after vaginal delivery, 714 after cesarean delivery, and 5 after a cesarean performed for a second twin. Women were eligible if they had singleton or multiple pregnancies, regardless of parity, delivered stillborn or live-born babies in a participating maternity unit, at or after a gestation of 22 weeks (or, in the absence of a specific date for the beginning of the pregnancy, birth of a child ≥500 g), and with an immediate PPH, defined as blood loss >500 mL in the 24 h after delivery. The subanalysis presented here excludes cesarean deliveries because in the HERA study the definition of PPH was >1000 mL for the cesareans.

### 2.3. Interventions

In each case of PPH, the medical data, including hemoglobin level, type of surgical treatment, and maternal outcomes, were recorded. Blood loss was to be estimated at least visually, but the habitual method used in each unit and specified in each woman’s records was recorded. Professionals in each unit collected data prospectively for 6 months, entering them onto electronic case report forms via a secure website.

### 2.4. Measurements

In this study, hemoglobin results had to be available from both the antepartum and the immediate postpartum period (the lowest available level and before any transfusion), together with the dates the samples were taken. The antepartum hemoglobin results had to date from less than 4 months (maximum 130 days) before delivery. In France, a hemoglobin assay is part of the statutorily required 6th month laboratory work-up, and another is required for the anesthesia consultation mandatory in the 8th month at the maternity ward where the woman plans to give birth [27]. The latter consultation routinely includes a complete blood count and coagulation testing. The 2015 French guidelines define postpartum anemia as hemoglobin < 110 g/L at 48 h after delivery [28]. This should be checked only among women with bleeding or symptoms of anemia after giving birth, ideally at 48 h postpartum. For this study, we excluded women for whom the delta hemoglobin was negative (*n* = 39), those for whom the exact blood volume of loss was unknown (*n* = 75), those for whom the date of blood sampling was inconsistent (*n* = 9), and those for whom one of the two hemoglobin assays was missing (*n* = 401). Finally, the study sample comprised 2964 women.

### 2.5. Outcomes

The primary outcome was defined by the hemoglobin loss in g/L (antepartum hemoglobin minus postpartum hemoglobin). The secondary outcomes were: hemoglobin loss according to the severity of PPH (mild or severe PPH), and the sensitivity, specificity, false positive and false negative values for 3 hemoglobin reduction thresholds selected from the literature (≥20 g/L, ≥40 g/L, and >10%) to optimize the identification of women who have had a postpartum hemorrhage [18,19,22,29,30].

### 2.6. Analysis

The medical data for all women with a PPH and according to its severity were described. Data for women with mild PPH were compared with those for severe PPH. The overall hemoglobin reduction for all women was calculated as a percentage, mean decrease, and median (with the interquartile range: IQR) decrease, and these were compared by level of PPH severity.

The descriptive results are expressed as percentages. The χ^2^ test (or Fisher’s exact test when appropriate) was used to compare the qualitative (categorical) variables and Student’s t test for the quantitative (continuous) variables. The sensitivity, specificity, and positive and negative predictive values were calculated with their 95% CIs. Because our population included only women with PPH, we simulated a mean change in the hemoglobin of women who gave birth without a hemorrhage from the data in the literature [19].

The analysis was performed with SAS software (SAS v9.4, SAS Institute Inc., Cary, NC, USA). A *p* value < 0.05 was considered significant.

## 3. Results

### 3.1. Characteristics of the Study Subjects

Table 1 describes the medical characteristics of the women included in this study. Our sample was composed principally of women with singleton pregnancies (96.9%); their mean age was 29 ± 5.4 years (Table 1). The most common reason for the PPH was uterine atony (58.7%) with a mean blood volume loss of 913 ± 471 mL (Table 1). In all, 13.6% of patients had a blood transfusion, and 3.1% had a vascular embolization.

These data differed between the groups of women with mild and severe PPH. We noted lower subjective estimates of blood loss, fewer vascular embolizations, and fewer blood transfusions in the group with mild PPH than in those with severe PPH; these results were, respectively, 19.0% vs. 24.7% (*p* < 0.0002), 1.3% vs. 5.2% (*p* < 0.0001), and 5.6% vs. 22.4% (*p* < 0.0001) (Table 1).

### 3.2. Main Results

The mean antepartum hemoglobin was 119 ± 11 g/L and did not differ significantly between the mild and severe PPH groups (*p* < 0.67) (Table 2). The mean change in hemoglobin was 30 ± 14 g/L and was significantly higher in the group with severe PPH than in the group with mild PPH (33 ± 15 g/L vs. 27 ± 13 g/L, *p* < 0.0001) (Table 2). Globally, hemoglobin decreased by at least 10% in 90.4% of the entire cohort, by ≥20 g/L among three quarters of the cohort, and by ≥40 g/L in only one quarter (Table 2). These diagnostic thresholds were exceeded significantly more frequently in cases of severe PPH (*p* < 0.001) (Table 2).

Looking at the diagnostic performance of the hemoglobin thresholds selected (decreases exceeding > 10%, ≥20 g/L, and ≥40 g/L), we observed that all sensitivity and specificity values were <65%, the positive predictive values were between 35% and 94%, and negative predictive values were between 14% and 84% for all PPH (Table 3). The positive likelihood ratio was less than 2 and the negative likelihood ratio was less than 1. These values barely changed at all when assessed by the level of PPH severity (mild or severe) (Table 3).

## 4. Discussion

The mean change in hemoglobin was 30 ± 14 g/L among all women with PPH. A hemoglobin decrease >10% was observed in 90.4% of them. Decreases of ≥ 20 g/L and ≥40 g/L occurred in respectively 73.9% and 23.7% of the cohort. The mean hemoglobin change and the preceding selected thresholds of hemoglobin decrease were always higher in the group with severe compared with mild PPH (*p* < 0.0001) (Table 2). To identify a PPH > 500 mL, the values for sensitivity and specificity were <65%, while positive predictive values were between 35% and 94%, and negative predictive values were between 14% and 84%. The positive likelihood ratio being less than 2 and the negative likelihood ratio being less than 1 show that the diagnostic contribution of hemoglobin measurements is quite poor. These values barely improved at all when assessed by level of PH severity (mild or severe) (Table 3).

The first strength of our study is that it is a multicenter prospective population-based cohort study of women with PPH. Its second strength is its size (*n* = 2964 PPH).

Determining the precise quantity of blood loss during delivery continues to be a challenge for perinatal practitioners. As studies have shown that the visual estimation of blood loss is imprecise and subjective [15], some authors consider that the before/after delivery delta hemoglobin is useful [18,19]. In the retrospective study by Charafeddine et al. (*n* = 407 vaginal deliveries without PPH), hemoglobin decreased >20 g/L for 54 women, which was interpreted to indicate a 13.3% rate of undiagnosed PPH [18]. They reported a median hemoglobin level of 123 g/L ± 6 at entry into the labor room and 117 ± 7 at 48 h postpartum. In our PPH cohort, antepartum hemoglobin was 119 ± 11 g/L and the lowest hemoglobin was after PPH, at 89 ± 15. The latter was lower than in the study by Charafeddine et al., but our cohort included only deliveries with PPH. In the prospective by Girault et al. testing the impact of controlled cord traction on PPH incidence (*n* = 3917), 11.2% of the women had an undiagnosed abnormal postpartum blood loss (UPPBL) after vaginal delivery (peripartum delta hemoglobin ≥ 20 g/L) [19]. In that study, the median peripartum hemoglobin changes were 250 g/L (IQR, 22–30) for the UPPBL group, 24 g/L (IQR, 15–33) in deliveries with diagnosed PPH, and 5 g/L (IQR, 0–11) in the control group (deliveries with neither UPPBL nor PPH). The median peripartum change in hemoglobin in our study was higher than in theirs: 29 g/L (IQR, 19–39). Our study had more than 6 times more deliveries with PPH: 2964 in our study vs. 430.

Atukunda et al. studied the women included in a randomized controlled trial to test PPH prevention (oxytocin vs. misoprostol) [29]. They found a lower mean hemoglobin change than we did: from 10 ± 10 g/L with a decrease exceeding 10% in only 22.6% of the women. We must note that only 258 of 1140 deliveries in their study had a PPH.

Anger et al. simultaneously analyzed a study in Pakistan into preventing PPH (*n* = 1058) and two separate studies about treating it (*n* = 1283). Because they estimated hemoglobin levels with the HemoCue device, which has been shown to give higher readings than automated laboratory methods, postpartum hemoglobin levels may have been somewhat underestimated. In all three studies, most women with a severe PPH showed a hemoglobin decrease of ≥20 g/L: 68% in the Pakistan study and 63% in the multicountry trials [22]. Inversely, for mild PPH, in both the Pakistan study and the multisite trials, fewer than half showed a hemoglobin decrease of ≥20 g/L (respectively, 31% and 42%) [22]. In our study, a hemoglobin decrease ≥ 20 g/L was observed for 68.0% of the women with mild PPH and 80.2% of those with severe PPH.

The sensitivity of a hemoglobin decrease of >10% or ≥20 g/L was better among the women with severe PPH than those with mild PPH; respectively, 77.9% vs. 66.4%, and 48.9% vs 47.3%. This result was not observed for a hemoglobin decrease of ≥40 g/L (Table 3). The specificity of a hemoglobin decrease was higher at the thresholds ≥ 20 g/L and ≥40 g/L for severe PPH compared with mild PPH—respectively, 72.1 vs. 64.2%, and 66.1 vs. 58.7%. This was not, however, the case for the endpoint “hemoglobin decrease > 10%”. The highest positive predictive value was observed for a hemoglobin decrease > 10% and the lowest for a decrease ≥ 40 g/L. The highest negative predictive value was noted for a hemoglobin decrease ≥ 40 g/L and the lowest negative predictive value for a decrease > 10%.

In a prospective cohort study of 634 women who had vaginal deliveries, Palm et al. found sensitivity values (22.3% to 45.5%) that were highest for the lowest hemoglobin threshold chosen on the third day postpartum (<80 g/L, <90 g/L, <100 g/L, <110 g/L) [20]. Inversely, the specificity (88.8% to 93.2%) and positive predictive values (6.7% to 61.3%) both rose with the hemoglobin level [20]. The sensitivity and positive predictive value were both the best among deliveries with a blood loss ≥600 mL, especially for hemoglobin values < 80 and <90 g/L—respectively, 87.5% vs. 67.6%, and 89.8% vs. 90.9%. Nonetheless, the positive predictive values remained low: 16.5% for hemoglobin <80 g/L and 29.5% for hemoglobin < 90 g/L. Palm et al. concluded that only a minor proportion (≤14%) of the variation in hemoglobin on the third day after delivery was explained by the quantity of blood loss [20].

Steele et al. studied the utility of routine postpartum hemoglobin assessment in a before-after study (*n* = 800) [31]. They concluded that eliminating routine complete blood count testing was associated with a decreased transfusion rate (5.5% vs. 1.8%; *p* = 0.007) despite similar transfusion risks. The complete blood count decreased from 59.0% to 22.2% (*p* < 0.0001). No adverse bleeding outcomes occurred during the after period. The targeted blood count policy resulted in lower costs and improved the quality of patient care [31].

Our study has several limitations that warrant discussion. The first limitation is that our database did not include women without PPH, thus preventing the analysis of correlations between the blood volume loss and the hemoglobin value after delivery. We thus used data from a French prospective study [19] conducted in 2010–2011 to calculate the accuracy of the test (sensitivity and specificity) and determine how it performs in the population tested (predictive values and likelihood ratios). The second limitation is that we excluded from our analysis women with hemoglobin values higher after than before delivery (*n* = 39 over 3488 eligible women for the study: 1.12%). Palm et al. noted that more than 30% of all women had hemoglobin values on the third day that were higher than before delivery, which is perhaps explained by increased hematocrit levels after vaginal delivery. The change in hemoglobin concentration from antepartum to postpartum especially underlines the change in plasma volume. Elsewhere, it has been reported that the lowest level of hemoglobin concentration is observed during the 6-week postpartum period, that is, after hospital discharge [32]. The last limitation of our study is that the study did not standardize for the amount of fluid replacement after the delivery, which can result in hemodilution. This information is not available in our database. However, while the amount of fluid replacement during and after the delivery cannot be standardized, French guidelines, issued in 2004 and updated in 2014, govern the management of patients with PPH, and are applied in clinical practice similarly for all women with a PPH [28,33,34].

## 5. Conclusions

In conclusion, the mean hemoglobin loss was 30 ± 14 g/L among women with any PPH. The identification of women with one yielded values for sensitivity and specificity < 65%, positive predictive values between 35% and 94%, and negative predictive values between 14% and 84%. The positive likelihood ratio was less than 2 and the negative likelihood ratio less than 1. Accordingly, the determination of hemoglobin change between before and after delivery should not be used as a routine PPH diagnostic screening test for all vaginal deliveries.

This test should be reserved for women without a diagnosis of PPH during the first 24 h (or 48 h) postpartum who have clinical signs of anemia or for women with a known PPH, in order to be able to adapt medical support for them during the three days after delivery. The proper and timely diagnosis of immediate PPH should include a meticulous estimation of blood loss before the patient’s clinical signs change. The visual estimation of blood loss can be improved by simulating clinical scenarios with known measured blood loss and by using collector bags (or weight of blood lost). It is important to support a patient blood management policy during pregnancy, childbirth, and postpartum for all women during this period, as in other surgical and medical specialties, to improve pregnancy outcomes and optimize resources [35]. New research should assess the implementation of patient blood management in the field of obstetrics, and the efficacy, effectiveness, and utility of the shock index (ratio of heart rate divided by systolic blood pressure), in a randomized clinical trial.

## Figures and Tables

**Table 1:** Women’s characteristics overall and by severity of the postpartum hemorrhage.

Deliveries with PPH	Global PPH ^1^ *n* = 2964 % [Mean ± SD]	Mild PPH ^2^ *n* = 1548 % [Mean ± SD]	Severe PPH ^3^ *n* = 1416 % [Mean ± SD]	*p* Value ^4^
**Singletons**	96.9	97.7	95.9	0.004
**Women’s age**	*n* = 2921	*n* = 1528	*n* = 1393	
	[29.0 ± 5.4]	[28.7 ± 5.3]	[29.3 ± 5.4]	0.001
<18 years	0.8	0.9	0.7	0.08
≥18–<35 years	83.8	85.1	82.4	
≥35 years	15.4	14.1	16.9	
**PPH causes**				
Uterine atony	58.7	56.3	61.3	0.005
Placental retention	38.8	37.1	40.7	0.04
Vaginal and/or perineal lacerations	25.2	25.7	24.7	0.5
Episiotomy	19.9	20.9	18.9	0.2
Placenta previa	2.1	1.7	2.5	0.1
Uterine rupture	0.4	0.2	0.6	0.08
Cervical lacerations	3.9	4.0	3.7	0.6
Vaginal wall hematoma	1.4	1.3	1.5	0.6
Other ^5^	2.0	1.8	2.2	0.4
**Total estimated blood loss (mL)**	[913 ± 471]	[715 ± 106]	[1129 ± 602]	<0.0001
**Blood loss estimations ^6^**	*n* = 2953	*n* = 1544	*n* = 1409	
Bag and/or aspiration and/or drains	91.1	92.1	90.0	0.04
Weighed	16.0	13.8	18.4	0.0007
Subjective measurement	21.7	19.0	24.7	0.0002
**Second-line pharmacological treatment**	*n* = 2954	*n* = 1542	*n* = 1412	
Prostaglandin	34.9	27.7	42.7	<0.0001
Recombinant factor VII	0.6	0.2	1.0	0.006
Fibrinogen	3.1	0.7	5.7	<0.0001
Iron infusion	27.3	20.0	33.3	0.3
Tranexamic acid	5.5	2.9	8.3	<0.0003
Other	3.8	3.4	4.2	0.3
**Second-line** **nonpharmacological treatment**	*n* = 2928	*n* = 1529	*n* = 1399	
Intrauterine balloon	0.9	0.1	1.8	<0.0001
Vascular embolization	3.1	1.3	5.2	<0.0001
Surgical acts	43.2	43.5	42.8	0.7
B-Lynch	0.3	0	0.6	0.003
Cho	0.1	0	0.1	0.2
Hypogastric ligation	0.3	0	0.6	0.001
Other vessel ligations	0.5	0.3	0.9	0.04
Cervical suture	3.4	3.2	3.5	0.6
Suture, vaginal wound	40.4	41.3	39.3	0.2
Emergency hysterectomy	0.4	0.1	0.9	0.001
Other surgery	0.3	0.1	0.5	0.03
**Transfusion**				
Yes	13.6	5.6	22.4	<0.0001
Mean number units of packed red blood cells	[3.0 ± 2.0]	[2.3 ± 0.8]	[3.3 ± 2.1]	<0.0001
Mean number fresh frozen plasma	[1.3 ± 2.1]	[0.3 ± 0.8]	[1.5 ±2.3]	<0.0001

^1^ PPH > 500 mL. ^2^ Mild PPH: >500 mL and <1000 mL. ^3^ Severe PPH: ≥1000 mL. ^4^ Comparison between mild and severe PPH. ^5^ Other causes, including (*n* = 59): coagulation disorders (*n* = 10 including 6 after retroplacental hematomas), amniotic fluid embolism (*n* = 2), uterine inversion (*n* = 2), arteriovenous malformation (*n* = 1), bilateral hematoma of the broad ligament of the uterus with wound of the left uterine vein (*n* = 1), hemorrhagic afterbirth (*n* = 5), or not determined (*n* = 38). ^6^ The estimation of blood loss could require more than one method of measurement.

**Table 2:** Values of hemoglobin decrease, globally and by PPH severity.

	Global PPH ^1^ *n* = 2964 % [Mean ± SD] Median (IQR ^5^)	Mild PPH ^2^ *n* = 1548 % [Mean ± SD] Median (IQR ^5^)	Severe PPH ^3^ *n* = 1416 % [Mean ± SD] Median (IQR ^5^)	*p* Value ^4^
**Hemoglobin before delivery (g/L)**	[119 ± 11]	[119 ± 11]	[119 ± 11]	0.67
**Lowest postpartum hemoglobin (g/L)**	[89 ± 15]	[92 ± 14]	[86 ±16]	<0.0001
**Hb Change (g/L)**	[30 ± 14]	[27 ± 13]	[33 ± 15]	<0.0001
	29 (19–39)	26 (17–35)	32 (22–42)	
**Hb decrease > 10%**	90.4	87.9	93.0	<0.0001
**Hb decrease** ** ≥ 20 g/L**	73.9	68.0	80.2	<0.0001
**Hb decrease** ** ≥ 40 g/L**	23.7	17.5	30.5	<0.0001

^1^ PPH > 500 mL. ^2^ Mild PPH: >500 mL and <1000 mL. ^3^ Severe PPH: ≥1000 mL. ^4^ Comparison between mild and severe PPH. ^5^ IQR: interquartile range.

**Table 3:** Performances of the different changes in hemoglobin thresholds as a diagnostic test for PPH.

	Global PPH ^1^ *n* = 2964 % (95%CI)	Mild PPH ^2^ *n* = 1548 % (95%CI)	Severe PPH ^3^ *n* = 1416 % (95%CI)
**HB decrease ** ** > 10%**			
Sensitivity	61.1 (59.2–62.9)	66.4 (63.9–68.9)	77.9 (75.7–80.2)
Specificity	60.8 (55.2–66.5)	49.2 (42.0–56.4)	49.5 (39.7–59.3)
Positive predictive value	93.6 (92.4–94.7)	90.5 (88.7–92.3)	95.4 (94.1–96.6)
Negative predictive value	14.3 (12.3–16.3)	16.8 (13.6–20.0)	14.4 (10.7–18.1)
Positive likelihood ratio	1.6 (1.3–1.8)	1.3 (1.1–1.5)	1.5 (1.2–1.8)
Negative likelihood ratio	0.6 (0.5–0.7)	0.7 (0.6–0.8)	0.4 (0.3–0.5)
**HB decrease** ** ≥ 20 g/L**			
Sensitivity	59.4 (57.4–61.5)	47.3 (44.3–50.3)	51.8 (48.9–54.7)
Specificity	63.0 (59.6–66.4)	64.2 (60.0–68.5)	72.1 (66.9–77.4)
Positive predictive value	81.9 (80.0–83.8)	73.8 (70.5–77.1)	88.3 (85.9–90.7)
Negative predictive value	35.5 (32.9–38.0)	36.4 (33.2–39.6)	26.9 (23.8–30.1)
Positive likelihood ratio	1.6 (1.4–1.8)	1.3 (0.08–1.9)	1.9 (1.5–2.2)
Negative likelihood ratio	0.6 (0.5–0.7)	0.8 (0.7–0.9)	0.7 (0.6–0.7)
**HB decrease** ** ≥ 40 g/L**			
Sensitivity	61.7 (58.1–65.3)	55.0 (49.1–60.9)	54.9 (50.2–59.6)
Specificity	64.4 (62.4–66.3)	58.7 (56.0–61.4)	66.1 (63.1–69.0)
Positive predictive value	35.0 (32.4–37.7)	22.0 (18.9–25.2)	41.5 (37.5–45.6)
Negative predictive value	84.4 (82.7–86.1)	86.0 (83.7–88.3)	76.9 (74.1–79.8)
Positive likelihood ratio	1.7 (1.6–1.9)	1.3 (0.2–1.5)	1.6 (1.4–1.8)
Negative likelihood ratio	0.7 (0.6–0.7)	0.8 (0.7–0.9)	0.7 (0.6–0.8)

^1^ PPH > 500 mL. ^2^ Mild PPH: >500 mL and <1000 mL. ^3^ Severe PPH: ≥1000 mL.

## Data Availability

No data available.
